# Supplementary material for: Effect of pillow height on the biomechanics of the head-neck complex: investigation of the cranio-cervical pressure and cervical spine alignment
Source: PeerJ. 2016 Aug 31;4:e2397. doi: 10.7717/peerj.2397 (PMC5012320; doi:10.7717/peerj.2397)
Supplement: Data S1 [file peerj-04-2397-s001.pdf]

	Gender	Height (cm)	Weight (kg)	Neck Length (cm)
Subject 1	M	184	56	15.52
Subject 2	M	173	61	14.28
Subject 3	M	180	70	14.52
Subject 4	M	180	68.5	14.51
Subject 5	M	175	85	12.1
Subject 6	F	156	50	11.56
Subject 7	F	158	51	11.49
Subject 8	F	160	48	12.15
Subject 9	F	158	49	12.45
Subject 10	F	163	54	13

	Cranial Region							
	Average Pressure (kPa)				Peak Pressure (kPa)			
	H0	H1	H2	H3	H0	H1	H2	H3
Subject 1	5.56	5.24	4.14	4.37	15.53	13.21	10.42	10.67
Subject 2	3.46	4.13	3.91	6.3	7.87	12.18	8.81	15.23
Subject 3	4.14	4.47	5.47	5.65	10.97	11.66	11.42	15.74
Subject 4	5.32	4.6	3.98	5.58	18.08	9.49	11.06	22.31
Subject 5	3.02	2.91	3.37	6.15	6.32	6.27	10.51	15.58
Subject 6	3.52	4.6	5.73	6.62	8.31	14.47	15.38	21.35
Subject 7	4	3.36	4.58	5.69	11.38	8.07	13.63	15.92
Subject 8	4.87	4.67	6.21	7.06	13.87	14.23	14.67	16.32
Subject 9	3.13	4.71	5.28	5.45	11.33	10.32	11.88	9.26
Subject 10	4.91	6.46	6.13	6.38	14.22	18.23	14.64	17.89

	Cervical Region							
	Average Pressure (kPa)				Peak Pressure (kPa)			
	H0	H1	H2	H3	H0	H1	H2	H3
Subject 1	4.86	5.18	6.36	5.72	11.9	11.48	13.94	14.48
Subject 2	4.56	6.51	6.99	8.18	8.4	15.16	13.5	26.66
Subject 3	3.52	4.36	6.5	6.32	8.59	14.65	18.94	21.08
Subject 4	3.51	4.33	4.71	4.47	10.37	10.47	10.27	14.74
Subject 5	4.33	4.67	5.48	7.03	8.05	8.99	12.56	22.85
Subject 6	3.78	6.91	9.07	6.78	11.18	17.5	16.66	23
Subject 7	3.9	7.18	5.96	6.83	19.55	20.51	20.83	25.56
Subject 8	4.09	4.2	7.32	4.91	11.56	11.29	18.59	11.51
Subject 9	4.18	5.21	6.79	7.53	9.8	14.32	20.63	26.66
Subject 10	3.32	5.34	6.78	6.3	6.41	22.93	22.09	17.85
