# Supplementary material for: Effect of pillow height on the biomechanics of the head-neck complex: investigation of the cranio-cervical pressure and cervical spine alignment
Source: PeerJ. 2016 Aug 31;4:e2397. doi: 10.7717/peerj.2397 (PMC5012320; doi:10.7717/peerj.2397)
Supplement: Data S2 [file peerj-04-2397-s002.pdf]

Figure 6	Cranial Height (cm)			
	H0	H1	H2	H3
Subject 1	14.6175	11.73	19.5975	19.8075
Subject 2	15.09	25.5975	29.7975	31.8225
Subject 3	18.1875	23.52	26.67	30
Subject 4	19.56	22.8825	26.73	31.35
Subject 5	25.065	25.9425	28.785	29.1675
Subject 6	21.21	23.6325	25.9125	25.83
Subject 7	17.4975	21.9975	24.165	27.975
Subject 8	19.1175	23.0925	24.675	28.3725
Subject 9	20.3325	23.595	27.3375	27.5475
Subject 10	18.8925	22.5	24.6975	26.9625
Mean:	18.957	22.449	25.83675	27.8835
Standard Deviation:	3.003265	3.967704	2.83824	3.404374
Finite Element Prediction	21.28	21.59	22	22.43
